# Human Zinc Phosphide Exposure in Lebanon: A Case Report and Review of the Literature

**DOI:** 10.5811/cpcem.2020.10.47397

**Published:** 2020-12-01

**Authors:** Hani Hamade, Aynur Sahin, Carol Sukhn, Chady El Tawil, Jennifer Rizk, Ziad Kazzi, Tharwat El Zahran

**Affiliations:** * American University of Beirut, Department of Emergency Medicine, Beirut, Lebanon; † Karadeniz Technical University, Trabzon, Turkey; ‡ American University of Beirut, Department of Pathology and Laboratory Medicine, Beirut, Lebanon; § Emory University, Department of Emergency Medicine, Atlanta, Georgia, USA

**Keywords:** Zinc phosphide, metal phosphides, Lebanon, rodenticides

## Abstract

**Introduction:**

Toxicity from rodenticides such as metal phosphides is common worldwide, particularly in developing countries where consumers have access to unlabeled and uncontrolled insecticides and pesticides.

**Case Report:**

We present the first documentation of a metal phosphide exposure in Lebanon. A middle-aged woman presented to the emergency department following the ingestion of an unknown rodenticide. Spectroscopy analysis of the sample brought by the patient was used and helped identify zinc phosphide. The patient developed mild gastrointestinal symptoms and was admitted to the intensive care unit for observation without further complications.

**Review:**

We subsequently conducted a literature review to understand the diagnosis, pathophysiology, clinical presentation, and management of metal phosphide toxicity. Multiple searches were conducted on MEDLINE and PubMed, and articles related to the topics under discussion were included in the review. Metal phosphide is associated with significant morbidity and mortality involving all body systems. Patients presenting with metal phosphide intoxication need extensive workup including blood testing, electrocardiogram, and chest radiography. To date there is no antidote for metal phosphide toxicity, and management is mostly supportive. Many treatment modalities have been investigated to improve outcomes in patients presenting with metal phosphide toxicities.

**Conclusion:**

Emergency physicians and toxicologists in developing countries need to consider zinc and aluminum phosphides on their differential when dealing with unlabeled rodenticide ingestion. Treatment is mostly supportive with close monitoring for sick patients. Further research is needed to better understand metal phosphide toxicity and to develop better treatment options.

## INTRODUCTION

Rodenticides differ from one another in chemical formulations, mechanism of actions, and toxicity in humans. They contain many heterogeneous agents that are either organic (such as superwarfarins, strychnine, and sodium monofluoroacetate) or inorganic (such as arsenic salts, phosphorus, barium, and zinc/aluminum phosphide) compound groups.^1^ Metal phosphides, such as zinc phosphide (Zn3P2), are a significant cause of morbidity and mortality in developing countries where the use of these compounds is very common. Zinc phosphide is cheap, easily available, and a highly potent rodenticide.^2^ Mortalities due to accidental, suicidal, and homicidal exposure to zinc phosphide have been reported in Eastern countries such as India, Iran, Sri Lanka, and Thailand.^3^–^5^ Despite being associated with high mortality rates, there are currently no known antidotes for zinc phosphide toxicity, and the treatment is limited to supportive interventions.^6^ We report a case of zinc phosphide exposure in Lebanon, as well as a review of the existing literature on zinc phosphide poisoning.

## CASE REPORT

A 38-year-old female, known to have a history of generalized anxiety disorder and depression (on sertraline), presented to the emergency department (ED) with an intentional ingestion of an unknown black powder diluted in 450 milliliters (mL) of water, 30 minutes before arrival (Image). The powder was labeled as “rodenticide” and purchased from a local Lebanese market. The patient waited seven minutes after mixing the solution in a bottle and then drank 400 mL; she reported a garlic-like smell. Her other co-ingestions included three tablets of 0.5 milligrams (mg) alprazolam and two tablets of 500 mg acetaminophen. On arrival, she was alert and oriented, with normal vital signs (heart rate 77 beats per minute, blood pressure 122/81 millimeters of mercury (mm Hg), respiratory rate of 16 breaths per minute, pulse oximetry 98% at room air, and temperature of 37.2°C). Pupils were 4 mm and reactive bilaterally. Neurologic examination was normal with no rigidity or clonus.

**CPC-EM Capsule**

What do we already know about this clinical entity?

Metal phosphides are a significant cause of morbidity and mortality in developing countries. Toxicity frequently leads to shock and multiorgan failure.

What makes this presentation of disease reportable?

To report the first case of zinc phosphide exposure in Lebanon, as well as review the existing literature on zinc phosphide poisoning.

What is the major learning point?

Zinc and aluminum phosphides should be included on the differential when approaching an intentional ingestion of unlabeled product that is marketed as a rodenticide. Treatment is supportive with close monitoring for sick patients.

How might this improve emergency medicine practice?

Zinc phosphide is a potent rodenticide, and its distribution needs to be better controlled by officials to decrease potential accidental and homicidal toxic exposures.

The rest of her examination was unremarkable. Electrocardiogram was normal and chest radiography was unremarkable. Urine drug screen was detectable for acetaminophen but undetectable for amphetamines, barbiturates, benzodiazepines, cannabinoids, opiates, cocaine metabolites, tricyclic antidepressants, methadone, or phencyclidine. Her serum salicylate level was undetectable, and her acetaminophen level four hours post-ingestion was 18.6 mg per liter (L) (toxic level: >150 mg/L four hours post-ingestion). Initial laboratory results are displayed in the Table. Given that the ingestion of the unknown rodenticide was 30 minutes before presentation, activated charcoal with a dose of 1 gram (g)/kilogram (kg) was administered. The patient vomited once after the charcoal. The remaining 50 ml of the solution that the patient had ingested was sent for qualitative analysis. The sample was shaken and 10 ml was filtered on a 0.2μm filter, acidified, and run on inductively coupled plasma mass spectroscopy (Agilent ICP-MS 7500ce, Agilent Technologies, Inc., Santa Clara, CA) in a semi-quantitative mode for multiple metals. The method used was the EPA 200-7/8 M (US Environmental Protection Agency Method 200.7: Determination of Metals and Trace Elements in Water and Wastes by ICP-MS-atomic emission spectrometry). This test can detect many substances including anticoagulants, thallium, aluminum/zinc phosphide, and other heavy metals (aluminum, vanadium, chromium, manganese, iron, cobalt, nickel, copper, zinc, arsenic, selenium, strontium, silver, cadmium, barium, lead, mercury, and phosphorus).

The test detected zinc (14.7 mg/L, limit for quantification >0.005 mg/L) and phosphorus (14.7 mg/L, limit for quantification >0.005 mg/L). Other metals such as barium (0.026 mg/L), strontium (0.058 mg/L), manganese (0.036 mg/L), lead (0.024 mg/L), and thallium (0.007 mg/L) were detected minimally above the level of quantification (>0.005 mg/L) but not as high as phosphorus and zinc. Testing for whole blood lead and arsenic level were also performed and results were unremarkable. The patient was subsequently admitted to the intensive care unit for 24 hours where she remained hemodynamically stable and only complained of mild abdominal pain and vomited once. Serial coagulation profile was repeated and was within normal limits. The patient was later transferred to the psychiatric ward and discharged with no complications on day three of hospitalization.

## DISCUSSION

The above case highlights the challenges that emergency physicians and toxicologists can encounter in developing countries, where consumers have access to unlabeled and uncontrolled insecticides and pesticides. The differential diagnosis in the case of unlabeled insecticide toxicity is wide and includes toxicity by thallium, zinc and aluminum phosphide, long-acting anticoagulants, lead, or other heavy metals. Analysis of the exposure sample is a key step as it helps in identifying the active ingredient, allowing the medical team to make appropriate medical decisions. In the case under discussion, zinc phosphide did not lead to a significant morbidity or mortality. This may be due to the fact that the zinc phosphide powder was already unpackaged and wrapped in a newsletter at the shop prior to purchase. This form of storage leaves the substance exposed to air and humidity, resulting in the dissipation of the phosphine gas.^7^

There is a lack of updated evidence in terms of characteristics and management in zinc phosphide toxicity. Our goal was to provide an updated review of the literature in zinc phosphide toxicity, including important information on the pathophysiology, clinical presentation, diagnosis, and treatment. Since the toxic agent in zinc phosphide toxicity is phosphine, studies on phosphine and aluminum phosphide were also reviewed. We used the following search strategy: the National Library of Medicine’s MEDLINE database (PubMed) was systematically searched for articles from 1990 to date using the following keywords and strategy: 1) metal phosphide; 2) zinc phosphide; 3) metal phosphide; 4) toxicity or exposure or poisoning; 5) #1 AND #4; 6) #2 AND #4; 7) #3 AND #4; 8) antidote or therapy or treatment; 9) #5 AND #8; 10) #6 AND #8; 11) #7 AND #8; 12) phosphine AND toxicity AND poisoning AND treatment. Every search triggered further review of additional articles. Abstracts of articles were reviewed and selected for inclusion if they discussed metal phosphide toxicity or exposure and treatment. We also conducted an independent hand search of the bibliography of studies to identify relevant articles that were not identified on the initial automated search. No limits on types of articles were set. Articles were excluded by the senior and first author if found to be irrelevant to the scope of this review.

### Exposure

Zinc phosphide is an inorganic chemical compound that is found as dark gray or black powder.^8^ Upon ingestion, zinc phosphide is hydrolyzed by gastric acid, generating phosphine gas, which is rapidly absorbed through the gastric mucosa.^9^ In its purest form, phosphine is almost odorless, but its commercial grade has a disagreeable, garlic-like or decaying fish odor, due to impurities.^10^ The odor threshold for phosphine is 0.14–0.28 mg cubic millimeters (0.1–0.2 parts per million). However, odor is not always a reliable indicator of phosphine levels.^10^

Phosphine gas is absorbed through multiple routes (inhalational, dermal, and oral).^11^ It is worth noting that ingestion of the fresh rodenticide in the original packaging is associated with increased toxicity. Once the packaging is removed and the rodenticide exposed to moisturized air, it is rendered less toxic due to the conversion of the phosphide and dissipation of the phosphine gas.^7^

### Pathophysiology

Zinc phosphide is a highly toxic compound, and the ingestion of as little as 4–5 grams can lead to significant toxicity and death.^12^ The generated phosphine gas is widely distributed in the body to all organ systems.^13^ Phosphine is primarily eliminated through exhalation, but it is also oxidized and eliminated in the urine as phosphites.^14^ The pathophysiology of zinc phosphide toxicity is multifaceted, and multiple theories have been proposed to explain its toxicity. The phosphine gas inhibits the cytochrome C oxidase in the inner mitochondrial membrane, leading to dysregulation of the oxidative phosphorylation pathway.^15^ Additionally, nuclear and mitochondrial deoxyribonucleic acid damage through guanine oxidation is observed in the brain and the liver.^16^ Additionally, phosphine toxicity increases the production of oxygen reactive species, resulting in lipid peroxidation and tissue destruction, and ultimate organ collapse.^17^,^18^

### Clinical Characteristics

Patients with zinc phosphide intoxication usually have multisystem toxicity. The most common presenting signs and symptoms include nausea, vomiting, dyspnea, retrosternal or epigastric pain, and agitation, although patients may present in cardiovascular shock and hemodynamic instability.^3^,^19^ Zinc phosphide toxicity is associated with a high mortality rate, which can range from 37–100%.^20^

#### Gastrointestinal Toxicity

In a retrospective study conducted on 455 patients who had ingested zinc phosphide in Thailand, Trakulsrichai et al identified gastrointestinal symptoms as the most common presenting symptoms.^3^ However, gastrointestinal symptoms are also prevalent in patients who have been exposed through compound inhalation, as shown by Wilson et al.^19^ The common symptoms seen in cases of ingestion include nausea, vomiting, and abdominal pain.^4^,^19^ Phosphine is also associated with garlic-like breath.^11^ Additionally, zinc phosphide exposure is associated with hematemesis and corrosive changes in the esophagus and stomach of exposed patients.^20^–^22^ Esophageal strictures are another complication that develops following exposure, with cases of tracheaesophageal fistulas also reported.^23^–^25^

#### Cardiovascular Toxicity

Phosphine toxicity leads to a constellation of cardiovascular clinical effects. A collapse of the circulatory system is frequently observed, which typically results in refractory hypotension and shock.^26^ Tachycardia is a common finding, although in some cases bradycardia is observed despite the persistent hypotension.^27^ In a study of 25 patients with phosphine exposure, Kalra et al reported that phosphine toxicity leads to a specific set of hemodynamic changes, which include profound hypotension, decreased cardiac output, increased systemic venous pressure, normal pulmonary capillary wedge pressure, and inadequate systemic vasoconstriction.^28^ Patients with hypotension have been found to have left ventricular enlargement early on following intoxication, as well as hypokinesia of the left ventricle and septum, global hypokinesia, and in some cases akinesia.^29^–^31^

Additionally, studies investigating histological changes in cardiac tissue following exposure to phosphine confirmed the presence of myocardial edema with fiber separation, fragmentation of fibers, vacuolization of myocytes, as focal necrosis and neutrophilic and eosinophilic infiltrates in the heart.^32^–^35^ Autopsies of isolated cases have also demonstrated evidence of myocarditis, pericardial effusions, and pericarditis.^19^,^30^,^33^–^35^ Dysrhythmias including atrioventricular bundle branch blocks, atrial fibrillation, atrial flutter, and other supraventricular tachycardias has been observed in patients following exposure to phosphine.^36^ Additionally, ST-segment changes on the electrocardiogram (ECG) similar to those found in myocardial ischemia have been documented in some patients.^37^

#### Respiratory Toxicity

Labored breathing and chest tightness are among the most common symptoms in patients exposed to phosphine, particularly in those who inhale it.^6^,^19^ Coughing, cyanosis, and the presence of rales and rhonchi on auscultation are also frequent findings.^21^ Pulmonary edema and acute respiratory distress syndrome have also been reported in multiple studies.^38^,^39^

#### Central Nervous System Toxicity

Neurological signs and symptoms of phosphine toxicity include headache, dizziness, drowsiness, paresthesia, intention tremors, weakness, fasciculations, and altered mental status.^19^ Delayed symptoms may include seizures, delirium, and coma, all of which may be caused by electrolyte abnormalities as well as neuronal damage.^19^

#### Hepatic Toxicity

Elevation of transaminases and, to a lesser extent, jaundice can be observed in patients with phosphine poisoning.^7^,^19^ Saleki et al studied the histological findings in the livers of 33 deceased patients and noted that sinusoidal congestion (77.4%) and fine vacuolization of hepatocytes (71.1%) were the major findings following exposure.^40^ Cases of hepatic failure and hepatic encephalopathy have also been reported.^41^,^42^

#### Metabolic Findings

Metal phosphide toxicity is associated with glycemic derangement. Severe and persistent hypoglycemia is a common finding of phosphide poisoning, and it is believed to result from impaired hepatic gluconeogenesis and glycogenolysis.^43^ However, hyperglycemia is also observed in rare cases and is associated with poorer outcomes.^44^ While the underlying causes of the elevated glucose are not fully understood, timely correction of the glucose level is recommended.^44^ Another very common finding is metabolic acidosis, which is often severe and associated with poor prognosis.^12^,^45^ Hypokalemia secondary to vomiting, and hyperkalemia secondary to acidosis and renal failure may also develop.^3^,^7^

## DIAGNOSIS

Laboratory confirmation of phosphine exposure is usually not required if it is a known exposure.^2^ Phosphine is not tested for in the blood due to rapid oxidization into phosphites.^46^ However, a multitude of tests can confirm phosphide exposure and are required for forensic investigation. Qualitative color tests can be used to detect phosphine in biological samples. Testing can be done using silver nitrate, mercury chloride, potassium permanganate, or mercury diethyliocarbamate, and can be performed on samples of expired air, stomach content, urine, or liver tissue.^2^ Testing with silver nitrite paper is the most commonly used technique.^2^ Silver nitrate paper turns from blue to black when exposed to phosphine, and can detect phosphine concentrations as low as 0.05 mg/L.^2^,^12^

When phosphide poisoning is suspected, it is important to obtain laboratory tests that can determine the severity of toxicity as well as the prognosis of the patient. This includes blood testing for complete blood count, electrolytes, glucose, arterial blood gases and pH, hepatic function tests, and kidney function tests.^2^ Additionally, chest radiography is required to rule out the presence of pulmonary congestion, and an ECG is needed to detect cardiac involvement.^2^ Once phosphide poisoning is suspected, a medical toxicologist should be consulted early on for advice and guidance in management.^2^

## TREATMENT

There is currently no known antidote for zinc phosphide toxicity. Management is generally supportive and needs to be initiated promptly. Patients who are symptomatic at presentation require observation in a monitored setting until they are back to baseline.^2^

### Gastrointestinal Decontamination

Activated charcoal could be considered for patients with oral ingestion of zinc phosphide within one hour if there is no contraindication.^2^,^7^,^12^ However, the use of activated charcoal or other compounds such as potassium permanganate in gastric lavage for metal phosphide poisoning may be of limited benefit due to the lack of molecular interaction between these compounds and the metal phosphide.^47^

### Blood Pressure Control

Hypotension is a common finding in patients with zinc phosphide intoxication that requires immediate attention. Patients should be initially treated with fluid resuscitation.^2^ However, hypotension is often resistant to correction by fluids and requires the use of vasoactive agents such as norepinephrine or phenylephrine.^48^ Marashi et al also suggested using hydroxyethyl starch, a colloid that causes intravascular volume expansion in the treatment of hypotension.^26^ Hydroxyethyl starch was successfully used in one case reported, although further evidence is needed to determine its impact.^26^,^49^ In refractory hypotension that is resistant to fluids and vasopressors, intra-aortic balloon pumps was used in isolated cases with questionable benefit.^50^

### Electrolytes and Blood pH Corrections

The metabolic acidosis observed following zinc phosphide toxicity is suspected to be the result of oxidative phosphorylation inhibition and severe tissue hypoperfusion secondary to hypotension and shock.^4^,^51^ Sodium bicarbonate (NaHCO_3_) is recommended by multiple sources for acidosis.^2^,^12^ However, Marashi et al and Boyd et al recommended that sodium bicarbonate should be reserved for patients in shock with blood pH lower than 7.0 49,52 In some cases, hemodialysis and peritoneal dialysis were used to correct severe acidosis and fluid overload.^4^

Conflicting evidence exists as to whether metal phosphide toxicity leads to hypomagnesemia or hypermagnesemia and whether supplementation can lead to better outcomes. Chugh et al conducted a randomized study on patients who had been poisoned by aluminum phosphide, in which a group of patients was given several doses of magnesium while another group acted as controls. The treatment group had a significantly better survival rate compared with the control group.^54^ However, the benefits of magnesium supplementation remain unclear.

Potassium disturbances should also be addressed and corrected, as they may predispose or worsen dysrhythmias.^7^

### N-acetylcysteine

A decline of antioxidant levels such as glutathione is documented in patients following metal phosphide poisoning.^55^ In a retrospective review of 100 cases of aluminum phosphide poisoning, Bhat and Kenchetty found that the use of N-acetylcysteine (NAC) is associated with lower mortality rate, shorter hospital stay, and lower peak levels of aspartate and alanine transaminases.^56^ Tehrani et al conducted a randomized clinical trial aimed at assessing the usefulness of NAC in the treatment of aluminum phosphide toxicity. The authors reported a mortality rate of 36% in patients who received NAC and 60% in patients who did not.^57^ These findings suggest that NAC may play a role in the treatment of phosphine toxicity. Additionally, Bhalla et al conducted an interventional study and found no difference in mortality rate between the group treated with NAC and the group that did not receive it.^58^ Further studies are therefore needed to confirm the role of NAC in the management.

### Extracorporeal Membrane Oxygenation

Extracorporeal membrane oxygenation (ECMO) is being explored as a treatment modality in the management of patients with aluminum phosphide toxicity. Mohan et al reported a series of seven patients with severe acidosis and refractory shock who were treated with ECMO, where five patients survived and had full recovery of left ventricular function.^59^ In another study involving 83 patients, Mohan et al found improved short-term survival in 15 patients who were on ECMO. Therefore, ECMO may play a role in the management of phosphide toxicity although further evidence is needed.^60^

### Hyperinsulinemia-euglycemia

High levels of insulin are believed to promote carbohydrate utilization instead of fats, which allows for better myocardial functioning.^61^ Hassanian-Moghaddam and Zamani evaluated two groups of poisoned patients and treated one group with high-dose insulin euglycemia protocol. Patients in the treatment group had significantly longer hospital stays and better survival compared to patients who received supportive treatment only.^62^

### Blood Transfusion

Rahimi et al used packed red blood cells (PRBC) in rats poisoned with metal phosphide and reported improved acidosis and overall survival.^63^ In their study, they infused 1.5 mL of PRBCs into the poisoned rats, one hour after intoxication with aluminum phosphide (4–15 mg/kg). PRBC infusion improved the acidosis, electrolyte disturbances, and plasma troponin levels besides reversing the ECG changes. One proposed mechanism is that increased PRBCs chelate toxic intermediates through phosphine-hemoglobin interaction and modulate acid-base disturbances.^63^

### Other Modalities

In another study, minocycline reversed ECG abnormalities, heart failure signs, and kidney injury in rats intoxicated with metal phosphides. The authors postulate that minocycline’s effects are due to its ability to improve mitochondrial function and inhibit apoptosis.^64^ Melatonin is another drug that is being explored as a treatment modality following phosphine toxicity. Hsu et al showed that melatonin increases glutathione levels and decreases lipid peroxidation in the brain in vivo and in vitro.^16^ Asghari et al conducted a similar study on rats and observed a decrease in the phosphine-induced oxidative damage to the heart in the group receiving melatonin.^65^ Additionally, Ahmadi et al studied the use of dihydroxyacetone (DHA) in phosphide poisoning in rats and found that DHA resulted in 100% survival and prevented cardiovascular abnormalities. The authors reported a 100% survival rate with improved ECG abnormalities and more stable hemodynamic status.^66^

Triiodothyronine, vasopressin, and milrinone are also being tested in animal models and show promising signs.^67^ Triiodothyronine is associated with decrease in cardiac dysfunction, oxidative stress, and apoptosis. Vasopressin is being shown to have cardioprotective effects and cause increase in adenosine triphosphate production. Milrinone causes a decrease in oxidative stress and apoptosis.^67^ Liothyronine and acetyl-L-carnitine have been studied for potential benefit for phosphide toxicity treatment.^67^ Based on in vitro studies, theoretically 6-aminonicotinamide showed protective activity in hepatocytes and boric acid may act as a trapping agent to trap phosphine, which can be excreted in urine.^67^ Further studies are needed to assess the role of these potential compounds in the management of phosphide toxicity.

## CONCLUSION

Emergency physicians and toxicologists in developing countries often face challenges when caring for patients who have been exposed to unlabeled and unregulated pesticides commonly found in the marketplace. Zinc and aluminum phosphides should be included on the differential when approaching an intentional ingestion of unlabeled product that is marketed as a rodenticide. This article is a comprehensive review of phosphide toxicity clinical presentation and approach to management. Toxicity affects multiple organ systems and frequently leads to shock and severe metabolic acidosis. Metabolic, cardiovascular, hepatic, and renal complications are common. There remains to be found a recognized antidote for metal phosphide toxicity, and treatment is mostly supportive.

## Figures and Tables

**Image:**
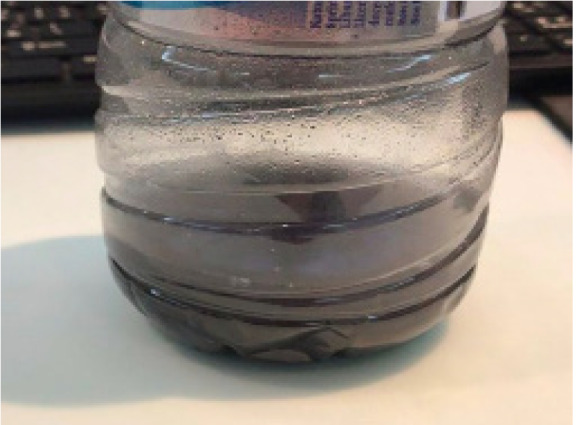
Residual sample (50 milliliters) of the diluted rodenticide ingested by the patient.

**Table:** Patient’s initial test results in the emergency department.

Test	Result	Reference values
White blood cells count	8200	4000 – 11000/mm^3^
Creatinine	0.8	0.5 – 1.0 mg/dL
Blood urea nitrogen	7	8 – 25 mg/dL
Bicarbonate	21	24 – 30 mmol/L
Aspartate transaminase	34	0 – 50 IU/L
Alanine transaminase	20	0 – 50 IU/L
Anion gap	12	
Activated partial thromboplastin time	26.3	27.0 – 39.0 sec
Prothrombin time	11	10.0 – 13.0 sec
International normalized ratio	1.1	0.9 – 1.2

mm^3^*,* cubic millimeters; *mg,* millligrams; *dL,* deciliter; *mmol,* millimoles; *L,* liter; *IU,* international units; *sec,* second.
